# Assessment of the predictive potential of cognitive scores from retinal images and retinal fundus metadata via deep learning using the CLSA database

**DOI:** 10.1038/s41598-022-09719-3

**Published:** 2022-04-06

**Authors:** Denis Corbin, Frédéric Lesage

**Affiliations:** 1 Laboratoire d’Imagerie optique et Moléculaire, Polytechnique Montréal, 2500 Chemin de Polytechnique Montréal, Montreal, QC H3T 1J4 Canada; 2 Institut de Cardiologie de Montréal, 5000 Rue Bélanger, Montreal, QC H1T 1C8 Canada

**Keywords:** Predictive markers, Risk factors, Biomedical engineering, Scientific data

## Abstract

Accumulation of beta-amyloid in the brain and cognitive decline are considered hallmarks of Alzheimer’s disease. Knowing from previous studies that these two factors can manifest in the retina, the aim was to investigate whether a deep learning method was able to predict the cognition of an individual from a RGB image of his retina and metadata. A deep learning model, EfficientNet, was used to predict cognitive scores from the Canadian Longitudinal Study on Aging (CLSA) database. The proposed model explained 22.4% of the variance in cognitive scores on the test dataset using fundus images and metadata. Metadata alone proved to be more effective in explaining the variance in the sample (20.4%) versus fundus images (9.3%) alone. Attention maps highlighted the optic nerve head as the most influential feature in predicting cognitive scores. The results demonstrate that RGB fundus images are limited in predicting cognition.

## Introduction

Individuals with mild cognitive impairment (MCI) are at greater risk of developing a form of dementia such as Alzheimer's disease^1^. A review of more than 30 studies approximated that 16.6% of the US population aged 65 and over had MCI^2^. Of this population, it is estimated that this form of cognitive decline is caused by Alzheimer's disease (AD) in more than 50% of cases, affecting more than 9 million people currently in the United States^3^. With increasing incidence with age (5.3% of people aged 65 to 74, 13.8% of people aged 75 to 84, and 34.6% of people aged 85 and older^3^) as well as an aging population, the proportional incidence of the disease in the population is anticipated to increase in the coming years, hence imposing significant burden and costs on the health care system^4^.

Distinguishing between cognitive decline and normal aging can be challenging. Thus, any form of cognitive decline must be diagnosed by a qualified clinician^5^. Cognitive tests are prioritized over imaging techniques such as amyloid PET or structural MRI to establish the diagnosis since they are cheaper, faster and easier to administer in a clinical context as in large scale studies. Furthermore, the added value of brain imaging to cognitive, neurological and psychiatric evaluations is limited as they do not currently improve the health prognosis of affected individuals^3^.

Cognitive tests take little to no time to administer (i.e., MoCA: 10 min or Mini-Cog: 3 min) providing the clinician a global view of the subject's cognitive abilities and are often enough to give a diagnostic. The MoCA cognitive assessment has been shown to detect MCI with high sensitivity and specificity^6^. However, there are currently no perfect tests that can detect all the intricacies of cognitive decline. Cognitive functions decline varies greatly across individuals, and different spheres of their cognition will be affected differently as they age or progress towards any form of dementia^7^. Furthermore, cognitive tests do not and cannot directly represent brain changes which are crucial in identifying those whose MCI is caused by AD^3^. Current treatments for AD are only symptomatic in nature^8^ since there is no method sensitive and specific enough to identify individuals in the earlier stages of the disease^9,10^. The lack of ability to effectively evaluate the cognition early in individuals may be one of the main barriers to building more clinical trials with potential disease modifying drugs. Subtle changes in certain areas of cognition as well as changes in the brain are therefore important to identify promptly for early diagnostic of Alzheimer’s diseases or MCI.

The retina is a window to the brain which allows the study of the central nervous system (CNS) without resorting to invasive nor expensive methods. According to previous evidence, physical changes such as a significantly thinner retinal nerve fiber layer^11–13^ and a lower vascular fractal dimension^14^ occur in AD and MCI individuals respectively. Furthermore, it has been shown that ex-vivo hyperspectral fundus images from Alzheimer's patients had observable amyloid aggregates which accompanied morphological and biochemical changes in the retina^15^. An increasing number of research papers are using the retina to target AD, MCI or cognitive functions for early diagnostic including hyperspectral imaging for amyloid detection in human^16,17^ and rodent models^15,18^, optical coherence tomography (OCT) for retinal layer thickness^13,19–24^ and RGB imaging of the fundus for vessel morphology^16,25–30^. A multimodal deep learning initiative has recently been successful in identifying AD stages with accuracy by combining brain volumes from MRI with clinical and genetic data^31^.

However, the studies cited above are limited by the small size of their dataset and by their uncommon approach (hyperspectral). In this work, retinal fundus features and metadata are exploited using a deep learning approach on the Canadian Longitudinal Study on Aging (CLSA)^32,33^ to establish the relation between cognition and the retina. By leveraging the large CLSA database (25,737 post-treatment fundus images), this study assesses whether an individual’s cognitive skills can be predicted from RGB retinal fundus images (a common imaging modality) and metadata using a deep-learning approach. The main contributions of our work include an investigation of the reliability of predicting age, blood pressure, body mass index (BMI), cognitive scores and APOE4 status from a multimodal deep learning approach and the first large-scale study combining fundus images and metadata to predict cognitive related abilities.

## Results

### Study population

Following all preprocessing steps, 14,711 participants with complete information were used from the CLSA dataset, totaling 25,737 fundus images. All individuals in the cohort were considered healthy based on the eligibility criteria of the biobank used for the project (CLSA aims to follow healthy individuals as they age over the next 20 years). With regards to retina-related diseases and according to the available information from CLSA, a minority reported being affected by glaucoma (3.86%) or macular degeneration (3.86%). Demographics of the individuals are summarized Table 1 and compared to similar studies in Table 2.

**Table 1:** Descriptive statistics of predicted variables for each subset of data.

Characteristics	Training set (n = 18,000)		Validation set (n = 3860)		Testing set (n = 3877)	
	Mean	Std.	Mean	Std.	Mean	Std.
Age (years)	60.93	9.57	60.93	9.46	61.02	9.54
Body mass index (A.U.)	28.06	5.44	27.91	5.19	28.14	5.50
Systolic BP (mmHg)	120.35	16.43	119.93	16.45	120.46	16.47
Diastolic BP (mmHg)	74.49	9.89	74.26	9.76	74.48	9.82
Executive function (A.U.)	42.72	12.45	42.86	12.58	42.84	12.61
Speed (A.U.)	59.42	14.42	59.40	14.14	59.40	14.84
Memory (A.U.)	53.93	15.73	53.80	15.69	53.95	15.97
Inhibition (A.U.)	60.78	11.83	60.98	11.63	60.87	11.72
Global cognition (A.U.)	38.97	11.86	38.98	11.74	39.01	12.20
Right eyes (%)	54.27	–	54.02	–	54.11	–
Male (%)	49.29	–	49.44	–	49.35	–

Significant values are in bold.

**Table 2:** Difference between CLSA subset and similar works aiming to predict cognition related measurements.

Characteristics	Dataset			
	CLSA (ours)	Venugopalan^31^	Thanh Duc^64^	Oyama^65^
Modality	Multimodal (Fundus and metadata)	Multimodal (MRI, EHR, SNP)	rs-fMRI	NIRS
Number of images	**25,737**	202	331	202
Age (years)	**60.9 (9.55)**	N/A	74.3 (4.70)	73.4 (13.0)
Sex (% male)	50.66%	N/A	49.54%	43.07%
Predicted variable(s)	Global cognition, inhibition, memory, speed, executive function, sex, APOE4, BMI, SBP, DBP and age	AD status	MMSE scores and AD status	MMSE scores

Values from cohorts other than CLSA are reported from corresponding papers. Mean and standard deviation are reported for numerical values.

Significant values are in bold.

Previous studies aiming to predict cognition related measurements were smaller thus making them more prone to biases and limiting their predictive power^34^. More variables are also inferred here when compared to similar studies.

### Prediction of physiological and cognitive variables

InceptionV3^35^, MobilenetV2^36^ and EfficientNet^37^ were empirically tested to determine which model was more suited for the task as showed in Table 3. Architectures were first tested on the predicted age as it is a variable that was predicted in all similar studies, thus making it a good benchmark to evaluate different architectures on a specific dataset. The EfficientNet architecture achieved considerably higher R^2^ and lower MAE compared to other models. R^2^ values and MAE obtained for the InceptionV3 were a little lower compared to Poplin et al. (R^2^: 0.74, MAE: 3.26) results on the UK Biobank dataset^38^. Results for the MobileNetV2 were also lower compared to the initial findings of Gerrits et al. (R^2^: 0.89, MAE: 2.78) on the Qatar Biobank^39^. Nevertheless, the EfficientNet model performed best on the CLSA dataset, making it the architecture used for ensuing investigations.

**Table 3:** Effect of architectures on the mean absolute error (MAE) and the coefficient of determination R^2^.

Metrics on CLSA	Baseline	Network		
		EfficientNet-b3^21^	InceptionV3^19^	MobileNetV2^20^
Used by for the same task		Corbin et al. (ours)	Poplin et al.^22^	Gerrits et al.^23^
Age: R^2^ (95% CI)	0.00	**0.778 (0.764–0.792)**	0.693 (0.678–0.706)	0.707 (0.687–0.726)
Age: MAE, years (95% CI)	7.19	**3.24 (3.16–3.34)**	3.72 (3.61–3.84)	3.68 (3.57–3.79)

95% on predicted age. CIs on metrics were calculated with 2000 bootstrap samples the size of the test set. Baseline metrics are from predicting the mean value for all individuals.

Significant values are in bold.

**Figure 1:**
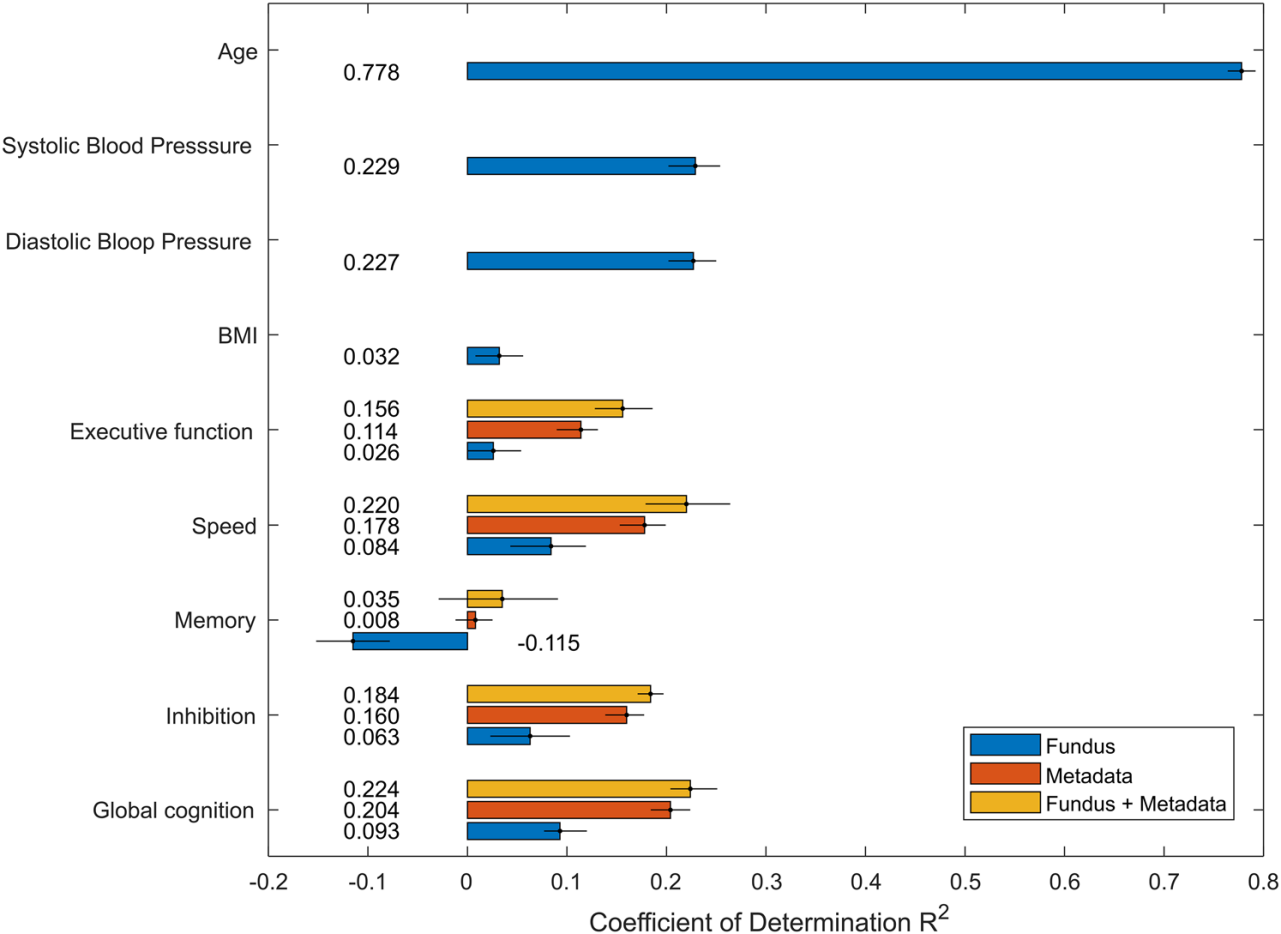
Predicted variables from regression tasks with corresponding coefficient of determination R^2^. Predictions are based on fundus image alone, on metadata alone or on fundus and metadata for cognitive scores. Error bars correspond to 95% CIs on R^2^ which were calculated with 2000 bootstrap samples.

Using the EfficientNet architecture, continuous variables were first predicted. R^2^ from fundus alone and fundus with metadata are presented in Fig. 1. First, the age (R^2^: 0.778 with 95% CIs 0.764–0.792 and MAE: 3.24 years with 95% CIs 3.16–3.34), SBP (R^2^: 0.229 with 95% CIs 0.202–0.254 and MAE: 10.94 mmHg with 95% CIs 10.62–11.27), DBP (R^2^: 0.227 with 95% CIs 0.202–0.250 and MAE: 6.80 mmHg 6.80 with 95% CIs 6.62–6.98) and BMI (R^2^: 0.032 with 95% CIs 0.008–0.056 and MAE: 3.99 with 95% CIs 3.86–4.14) were predicted. Variance explained by each model in this first round of experiments is on par with similar work from Poplin et al.^38^ and Gerrits et al.^39^ considering experimental differences. Averaging prediction on regression tasks like Gerrits et al. did not always lead to better R^2^ nor lower MAE. This may be explained by the fact that only 2 retinal scans (left and right eye) and sometime only one were available for prediction while the Qatar Biobank included 4 retinal scans per individual. Thus, reported regression results are based on fundus images rather than individuals.

In a second round of experiments, deep learning models were trained to predict cognitive scores. Cognitive scores were obtained by EFA followed by CFA. The CFA model was based on a higher order architecture with 4 first order latent variables (executive function, speed, memory and inhibition) and one second order variable (global cognition). EFA and CFA model development, analysis and validation is described in the Supplementary Information Fig. 1 and Tables 1, 2, 3, 4. Training was done by only using the fundus as an input, as in the first round of experiments. Performance metrics were weak for global cognition with a R^2^ of 0.093 (95% CIs 0.077–0.120), inhibition with a R^2^ of 0.063 (95% CIs 0.023–0.103), memory with a R^2^ of − 0.115 (95% CIs − 0.134 to − 0.078), speed with a R^2^ of 0.084 (95% CIs 0.043–0.119) and executive function with a R^2^ of 0.026 (95% CIs 0–0.054). Using only metadata, performance metrics improved achieving for global cognition a R^2^ of 0.204 (95% CIs 0.184–0.224), inhibition with a R^2^ of 0.160 (95% CIS 0.136–0.178), memory with a R^2^ of 0.008 (95% CIs − 0.012 to 0.025), speed with a R^2^ of 0.178 (95% CIs 0.153–0.199) and executive function with a R^2^ of 0.114 (95% CIs 0.090–0.131). Then, the effects of combining metadata with retinal fundus images were investigated. Global cognition could now be predicted with a R^2^ of 0.224 (95% CIs 0.204–0.251), inhibition with a R^2^ of 0.184 (95% CIs 0.171–0.197), memory with a R2 of 0.035 (95% CIs − 0.029 to 0.091), speed with a R2 of 0.220 (95% CIs 0.179–0.264) and executive function with a R2 of 0.156 (95% CIs 0.128–0.186).

**Table 4:** Hyperparameters used in the training of the proposed solution.

Hyperparameters	Value
Loss function	Mean absolute error (MAE) for regression task
	Binary cross entropy (BCE) for classification task
Optimizer	Standard gradient descend (SGD)
Momentum	0.9
Learning rate (LR)	0.001
Scheduler	Cosine annealing with 0.0001 max LR
Batch size	32
Epoch	100

**Figure 2:**
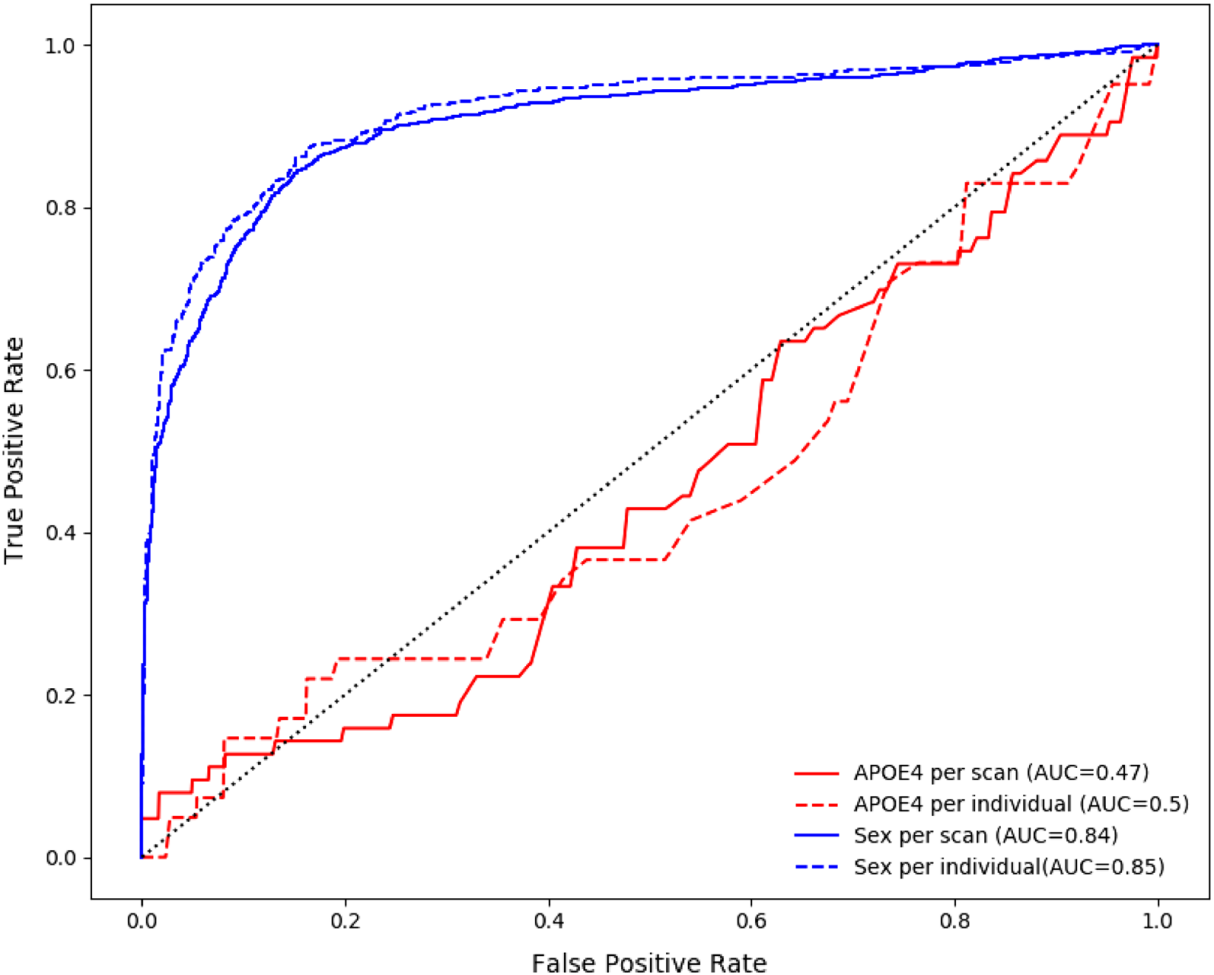
Predicted variables from classification tasks with corresponding AUC. Predictions are based on fundus image alone or on fundus and metadata. The effect of predicting at an image level versus an individual level is also illustrated.

ROC curves were then generated for binary variables like sex and APOE4 status, results are presented in Fig. 2. Combining retinal scans yielded marginally better results for binary classification problems. Models yielded a ROC AUC of 0.84 (95% CIs 0.830–0.857) for sex and 0.47 (95% CIs 0.397–0.544) for APOE4 status on an image level and a ROC AUC of 0.85 (95% CIs 0.842–0.871) for sex and 0.50 (95% CIs 0.397–0.544) for APOE4 status on an individual level.

### Investigation of attention maps

**Figure 3:**
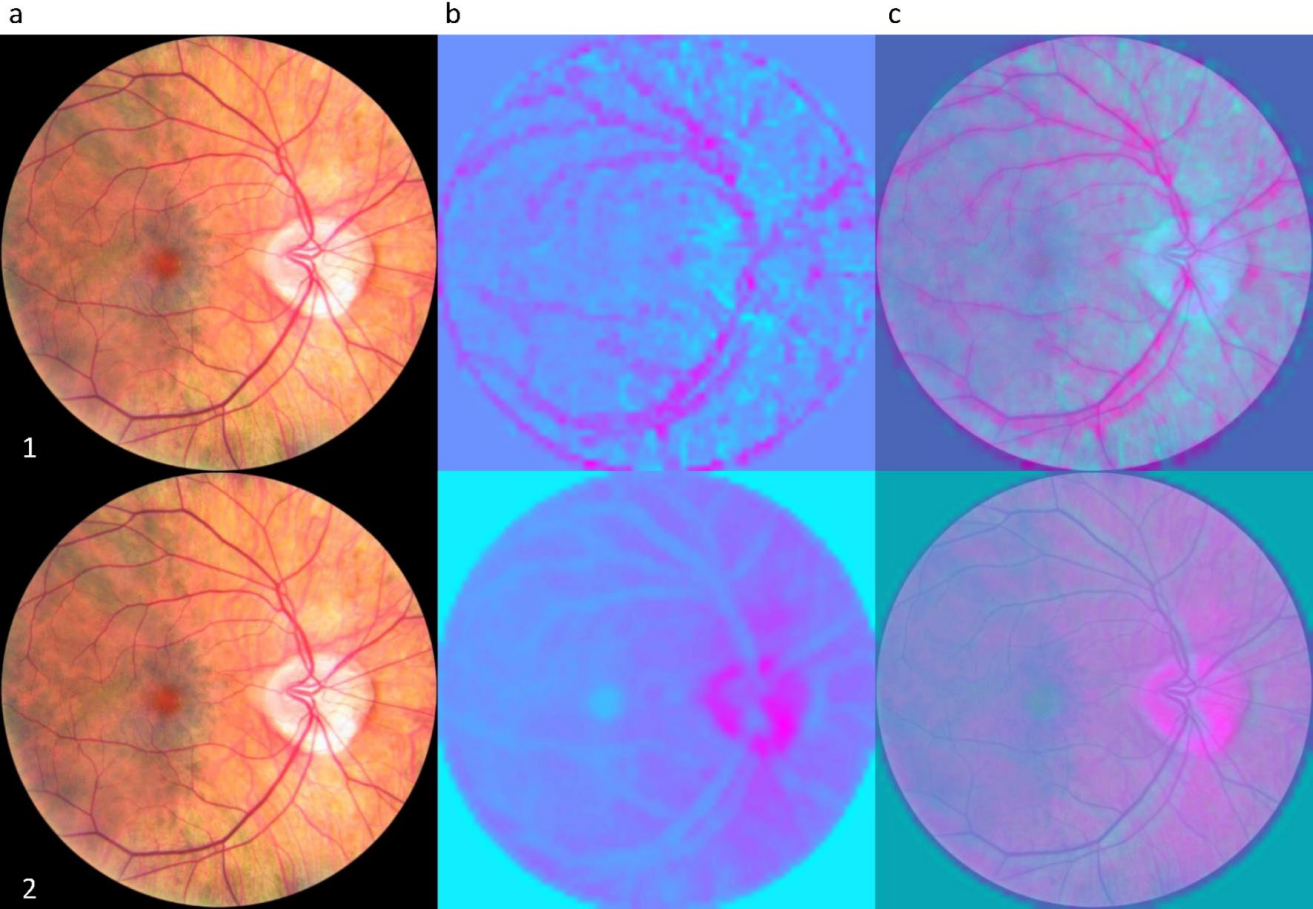
Saliency maps highlighting where the network focus when predicting age (1) and global cognition (2). Column (**a**) is the input image, (**b**) is the saliency map and (**c**) is the overlap of (**a**) and (**b**). The pink regions on the saliency maps are the one having influence in making the prediction while blue regions had lower importance. Highlighted regions were noted from 100 randomly selected images. For global cognition, the ONH was highlighted in 94% of sampled images, the background was highlighted in 44% of images, the vessels were highlighted in none of the images (0%) and non-specific regions (non-specific feature, edge of retinal scans, etc.) were highlighted in 24% of images. Regarding age, the ONH was highlighted in 1% of images, the background was highlighted in 2% of images, the vessels were highlighted in 89% of the images and non-specific regions were highlighted in 9% of images.

To further analyze the prediction of the model, attention maps, based on the methodology presented in^38^, were generated for some of the predicted. variable as shown in Fig. 3. Attention maps, also known as saliency maps, show where the network mainly focuses to make a certain prediction. It also allows to validate that those focused regions are not the same for every variable. It is important to know that the predictions are not all based on the same feature as it would mean they are highly correlated. Even though age and cognition are tightly linked, the generated attention map show that distinct regions were used for predicting those two variables. Based on randomly sampled images (n = 100) from the test set, the important features for predicting age are linked to the vasculature while for cognition, the optic nerve head (ONH), or the optic disc, was the main region of interest. APOE4 attention maps are not shown as they were inconsistent between individuals and highlighted nonspecific features, a consequence of poor performance for prediction APOE4 status in the first place.

Knowing from attention maps that vessels were important in predicting age, a pretrained segmentation network was used for vessel segmentation to explore the potential it had for classifying for age. For this regression task, the IterNet^40^ architecture was used as a backbone for feature extraction. The model was first pretrained on a private vessel segmentation dataset. Final convolution layers were then replaced with fully connected layers for the regression task. The backbone layers were first frozen for 400 epochs to let the fully connected part learn and then all layers were unfrozen for the last 100 epochs. While achieving a R^2^ of 0.667 and a MAE of 3.91 at age prediction, the network did not perform as well as the EfficientNet, but it confirmed that saliency maps can yield good insight into proposing new solutions for deep learning architecture.

## Discussion

Physiological variables such as age, blood pressure and body mass index were initially predicted to validate our approach by comparing to similar studies. We achieved a higher R^2^ compared to Poplin et al. for age (0.78 vs 0.74), but lower R^2^ for systolic blood pressure (0.23 vs 0.36), diastolic blood pressure (0.23 vs 0.32) and BMI (0.03 vs 0.13). However, it should be noted that while obtaining a lower R^2^ for SBP, we achieved a lower MAE of 10.94 mmHg compared to 11.35 mmHg as reported by Poplin et al. Since both cohorts do not have the same distribution of data, it is hard to compare both approaches. Poplin et al. superior R^2^ could be explained by the fact that they had a wider range of SBP thus allowing the network to better learn the associated features. Another factor that should be considered is that we filtered out more data compared to them (58% vs 12%) from preprocessing steps, hence lowering the size of our dataset. However, this was a necessary step in our case since only English-speaking individuals with complete metadata, physiological, cognitive and genomic data had to be kept in the dataset. This step could have lowered the variance in the dataset which might have limited the feature extractions abilities of our trained networks. As a second point of reference, results were compared to those reported by Gerrits et al. on the Qatar Biobank. We achieved slightly lower R^2^ for age (0.78 vs 0.89), SBP (0.23 vs 0.4), DBP (0.23 vs 0.24) and BMI (0.03 vs 0.13) compared to their results. Even though reported R^2^ are lower compared to Gerrits et al., 95% CIs largely overlapped for BMI and DBP suggesting no significant difference in predictive power for these two variables. It must be considered that their study had up to 4 retinal images per individual that were all acquired by the same camera at the same location. The study was also based on only 3000 individuals with similar background. Furthermore, the data primarily came from a Middle Eastern population which improves the predictive power of their algorithm on this specific subset of the population. Nevertheless, the proposed method as reported results are similar to previous studies thus validating our methodology. The R^2^ metric is variance dependant and may not be meaningfully comparable across different datasets.

Cognitive scores, imputed from the proposed CFA model (Fig. 1 in the Supplementary Information), were then predicted. Results showed that the executive function, speed, inhibition and global cognition could be predicted with low confidence (R^2^ < 0.1) while memory could not be predicted at all from retinal fundus images alone indicating a poor fit from the model. A negative R^2^ for memory means that the model was not able to learn how memory is represented in retinal fundus images. Hence, it also means that the model fits the data worse than a horizontal line representing the average of the dataset. However, adding metadata to the network as an auxiliary input to improve explained variance (R^2^) yielded better results compared to prediction made from fundus alone or metadata alone. This highlights the importance of metadata in predicting cognitive variables, and potentially the limitations of fundus towards brain evaluations. Indeed, while it is possible to explain some of the variance in the data from retinal fundus images, the metadata proved to be much more discriminatory. For cognition, metadata explained 20.4% of the variance alone, while retinal images were limited to explaining only 9.3% of the variance. The combination of approaches increased the variance explained by the model to 22.4%. These results point to the fact that metadata are more important than retinal fundus images in predicting cognition related variables. The metadata used (i.e., type of drinker, type of smoker, level of education achieved, perceived mental state, etc.) are also modifiable risk factors associated with Alzheimer’s disease and cognition^3^. Our observations are therefore consistent with previous observations from other studies that demonstrate that cognitive decline can be prevented/limited by addressing these modifiable risk factors. Also, we see that fundus images alone contribute, even if their contribution is small, to determine the cognition of an individual. This is consistent with the accepted theory that cognitive decline and Alzheimer manifest itself in the retina under different forms and that features in the retina may be used as potential biomarkers^41–45^. By extracting attention maps, it was found that attention was directed toward the optic nerve head to make predictions. This might be hypothetically explained by the fact that this densely populated zone by neuronal cells is the most susceptible zone at representing changes to the CNS like neuronal cell deaths. However, further investigations based on RGB retinal fundus are warranted to explore the influence of other contributing features of the retina on accessing cognitive decline/impairment.

Sex and APOE4 status were then predicted from retinal fundus images. Sex prediction yielded an AUC of 0.85 which is lower compared to the 0.97 reported in similar studies^38,39^. About the APOE4 status, the hypothesis was that it would be possible to identify APOE4+ individuals from fundus images since these individuals are more likely to have beta-amyloid deposits^46^. Knowing that these deposits can be imaged in the retina and that they scatter light in a distinct way^47^, it was assumed that it would be possible to identify these individuals using our classification algorithm. However, the classifier only reached an area below the ROC curve of 0.5 on the test set. Lack of success in identifying APOE4+ individuals might be attributed to their low representation in the dataset (1.55% of the sample size). This led to quick overfitting on the training set while remaining random on the validation and test set. Reducing the size of the network (less layers usually lead to less ability to learn specific pattern which contribute to overfitting), weighting the loss function based on sample weight and over/under sampling methods did not improve overfitting^48^. On the other hand, it is possible that RGB retinal fundus images might not be adequate in identifying small deposits that scatter lights differently as it is unlikely that this type of imagery is sensible enough to detect such small variations. Consequently, it would be interesting to perform the same type of large-scale analysis using retinal hyperspectral images since previous research has shown success in identifying Aβ+ patients using this modality in a smaller cohort^16,17^.

The main contribution of this study resides in the fact that this is the first large scale investigation aiming to predict cognition related variables based on retinal fundus images and metadata. To our knowledge, this work makes us the first to closely link retinal features to cognition using a large-scale database such as CLSA. While results are not the most decisive, they highlight a potential relation between retinal fundus images, metadata and cognition. We are also the first to suggest the use of the EfficientNet architecture for retinal features extraction linked to cognition. As with MobileNetV2, EfficientNet is a light network that usually performs better than InceptionV3. However, we showed that EfficientNet was superior compared to MobileNetV2 on our data as it achieved better performance on our dataset. Lastly, we showed that attention map can provide good insight in identifying potential solutions for feature extraction in image related task.

Despite the encouraging results, this study has several limitations. First, our study is limited to a Canadian population and its risk factors. Enrolled participants also needed to be able to give consent, thus limiting the odds of having individuals with severe cognitive impairment. In fact, individuals participating in the CLSA dataset had to be considered healthy (eligibility criteria) as they will be followed over a period of 20 years. Additionally, a cohort with older individuals characterized by greater variance in cognitive scores would have been beneficial as it may have improved prediction of cognition variables from retinal fundus images. Although we report promising results for predicting global cognition and speed, 95% CIs were wide for most predicted variables demonstrating that the method is not necessarily the most accurate into identifying very low of very high level of cognition. A larger dataset with a broader representation of different levels of cognition may lead to more accurate predictions and smaller CIs. In addition, the cognitive tests used in the CLSA cohort are not specifically designed to identify MCI individuals like the MoCA or the MMSE. It would be interesting in future work to determine whether our predictions, based on cognitive values from our CFA model, are in agreement with MMSE and MoCA to assess cognition. It would also have been interesting to treat this task a classification task rather than a regression task if we had gold standard diagnosis data for MCI. Another limitation to this study is that most metadata, and even sex, were self reported. This might induce bias in the dataset as some false claims by individuals could affect the data. For further studies, we suggest the use of more than one dataset (i.e. combining the UK Biobank to the CLSA) as it would allow to assess the generalizability of these findings.

## Conclusion

A new study was conducted on the Canadian Longitudinal Study on Aging (CLSA) database. Thousand of retinal fundus images with associated metadata were leveraged from CLSA using a deep learning-based approach to predict cognition and its different spheres. To the best of our knowledge, this is the first major study of its kind. The proposed solution was able to explain 22.4% of the sample variance with respect to cognition. In addition, attention maps showed that the network focused primarily on the optic nerve area to make its predictions, which will certainly guide future research. The proposed approach is still limited by multiple factors. First, it would have been interesting to validate our cognitive scale with an accepted test such as MoCA and MMSE. In addition, the cohort consisted of mainly healthy individual as it was an inclusion criterion in order to participate in the CLSA. The next step would be to assess whether these cognitive measures predicted by the network can contribute as an additional input to an Alzheimer classifier for example. Nevertheless, the results demonstrate that RGB fundus images are limited in predicting cognition. More research will be needed to assess the full potential of the retina and its abilities to be used as an early screening tool for cognitive decline.

## Methods

### Study participants

Data from the Canadian Longitudinal Study on Aging (CLSA) was used in this study. The CLSA is a large-scale and long-term (over 20 years) study based on more than 50,000 individuals who were recruited between the ages of 45 and 85. The data acquisition protocols and questionnaires to which every participant gave their informed consent were approved by 13 research ethics boards across Canada (Simon Fraser University, University of Victoria, The University of British Columbia, University of Calgary, University of Manitoba, McMater University, Bruyère Research Institute, University of Ottawa, McGill University, The Research Institute of the McGill University Health Centre, University of Sherbrooke, Memorial University). The use of data and analyses for this study was reviewed and approved by both Polytechnique Montreal’s and CLSA ethics committee. All methods were performed in accordance with the regulations and guidelines required by the Canadian Institutes of Health Research (CIHR) and the Canadian Tri-Council Policy Statement: Ethical Conduct for Research Involving Humans. Consent forms, questionnaires and detailed protocols for data acquisition can be found on the CLSA website at http://www.clsa-elcv.ca. Data used in this work can be separated in 5 groups: retinal fundus, genetics, physical measurements metadata, questionnaire metadata and cognitive measurements. Paired retinal fundus imaging was performed using a Topcon (TRC-NW8) non-mydriatic retinal camera on over 30,000 participants. Fundus images consist of color pictures with a 45° field of view with a non-specific centering protocol. As for genetics, genotype data for 26,622 successfully genotyped CLSA participants was available across 794,409 genetic markers of the Affymetrix Axiom array also used by the UK Biobank^49^. Only single-nucleotide polymorphism (SNP) for the apolipoprotein E (ApoE) were of interest in this study as the APOE-ε4 variant is associated with increased risk of Alzheimer’s disease^46^. For physical measurements, the two main variables of interest were the resting heart rate and blood pressure (BP) measurements. Using a VSM BpTRU blood pressure machine, 6 serial BP measurements were obtained yielding systolic, diastolic and resting heart rate. The average of the 6 measurements was used in this study as it more reliably reflects the resting BP of the individual by eliminating the white-coat effect. Questionnaire’s data comprised of many yes and no questions and scale questions. Just to name a few, type of smoker, type of drinker, recent injuries (stroke, falls in the last 12 months), senses rating (hearing, sight, smell), aids for senses, income (personal and house), sex and education were all considered totaling a total of 47 answers. Cognitive measurements were an area of focus for this study. The CLSA participants were asked to complete the following cognitive tasks: Rey Auditory Verbal Learning Test (REYI: instant recall of 15 words and REYII: 5 min delayed recall of the same words), Animal Fluency Test (AFT: name as many animal in 60 s), Mental Alternation Test^50^ (MAT: Alternating number and letter of the alphabet), Event-based Prospective Memory Test (PMT), Prospective Memory Test (PMT), Victoria Stroop Neurological Screening Test^51,52^ (STP: Broken into three progressive subtasks: (1) Colored dots, (2) Common words printed in same colors as dots and (3) Color words printed in non-corresponding colors of ink), Controlled Oral Word Association Test (FAS: Broken into three subtasks: Name as many words starting with F, A and S) and the Choice Reaction Time Test (CRT: Press a key on a screen as quickly and accurately as possible in a 60 s experiment). An exhaustive description of administrated tests is available in^53^. Questionnaire data and part of the cognitive measurements were acquired throughout a phone interview when possible and the rest of the data was sampled in person at one of the 11 data collection sites across Canada.

### Preprocessing

The first preprocessing step asserted retinal image quality to exclude poor-quality images as they are not suited for automated analysis systems which are highly dependent on image quality. The method proposed in^54^ was used for accessing quality of retinal fundus. Their architecture combines multiple color-space version of the retinal fundus to access quality with fusion blocks integrating features and prediction at multiple level^54^. Outputs are classified in three categories: “Good”, “Usable” and “Reject”. Around 5000 retinal fundi associated with a “Reject” tag were discarded at this step.

The purpose of the next step was to remove data that explained the same phenomena and it was principally aimed to cognitive measurements. Following a collinearity analysis, cognitive variables that were correlated by more than 0.8 were removed from the study^55^. Only REYI and REYII were correlated above 0.8 with a correlation score of 0.976. REYII was arbitrarily removed from the study.

The next goal was to remove outliers in questionnaire, physical and cognitive data. To avoid removing outliers in each category which could remove important individuals showing a significant cognitive impairment in one domain, a multivariate outlier detection method was chosen. To identify incomplete or wrongly entered data in one field that would interfere with the distribution of data, the Mahalanobis distance (MD) was used—which require the inverse correlation matrix which cannot be calculated if the variables are highly correlated^56^, hence the last step. Therefore, a MD with a p < 0.01 was used to identify outliers, removing a total of 1674 samples from the dataset.

Next, one-hot encoding was used for categorical variables (metadata and genomics). The missing values were replaced by 0 to nullify their effect on the output.

The last preprocessing step aimed to remove biases in the dataset caused by language. As shown in^57^, language had a significant effect (p < 0.001) on all cognitive measurements except the PMT and CRT test leading us to remove French speaking individuals from the study.

### Factor analysis models

To establish a representative and objective scale of cognition, exploratory factor analysis (EFA) was first performed. The EFA was used to group variables into scales where each of these variables have similar variances. This rather exploratory step made it possible to explore the underlying theoretical structure of the data and thus outline latent variables for confirmatory factor analysis (CFA). The uniformity within each scale was validated using the standardized Cronbach's alpha. Scales with weak alphas did not explain the same phenomenon, therefore indicating that the underlying variables were not explaining the same domain of cognition. To build an objective scale representing different spheres of cognition, a model based on CFA was developed. The objective of CFA is to test whether the data correspond to the hypothetical model based on the accepted theory^53^ as well as the observations made in the EFA. A model is then obtained which makes it possible to impute latent variables (the different spheres of cognition and cognition itself). For the architecture of the CFA model, a higher order architecture rather than a bivariate architecture was chosen. In^58^, it was shown that both would be valuable. However, compartmentalized analyzes mathematically give an advantage to the bivariate model and this mathematical advantage is greater than the real advantage that this architecture provides. It is also less likely that the wrong model will be falsely accepted when higher-order architecture is used over the two-factor configuration^58^. Cronbach’s alphas were then computed to access internal coherence of scales, a value greater or equal to 0.7 shows acceptable coherence^59,60^. All structural equation modeling and internal coherence evaluations were done using SPSS Statistic and SPSS Amos 26.

### Deep learning model development

At first, three networks were trained on fundus images: InceptionV3^35^, MobilenetV2^36^ and EfficientNet^37^ to assess performance on the dataset. InceptionV3 and MobilenetV2 were previously used in^38^^,^^39^ to predict cardiovascular risk factor from retinal fundus images in the UK Biobank and the Qatar Biobank respectively. EfficientNet-B3 was empirically tested for its recent state-of-the-art results on the ImageNet dataset. Hyperparameters mentioned in^38^^,^^39^ were respectively used for InceptionV3 and MobilenetV2 while EfficientNet-B3 hyperparameters are presented in Table 4.

Networks were preloaded using ImageNet weights to speed-up training and final layers were replaced with fully connected nodes matching the size of the labels. (Table 5). A distinct network was trained for every predicted variable as it yielded better results. Depending on the predicted variable, “regression networks” for continuous variables (age, blood pressure, cognitive score and etc.) and “classification networks” for binary variables (sex and APOE4 status) were trained with according hyperparameters. To incorporate the one-hot encoded metadata into the final prediction, a subnet was added (Table 6) to the main architecture.

**Table 5:** Modified architecture of the main network.

Layer type	Output shape
Input—retinal fundus	(600, 600, 3)
Pretrained network (InceptionV3, MobileNetV2 or EfficientNetB3)	(1000)
Fully connected	(500) without metadata subnet (750) with metadata subnet
Batch normalization	
ReLU	
Dropout (p = 0.2)	
Output—fully connected	(1)

**Table 6:** Architecture of the metadata subnet.

Layer type	Output shape
Input—metadata	(303)
Fully connected	(500)
Batch normalization	
ReLU	
Dropout (p = 0.2)	
Fully connected	(250)
Batch normalization	
ReLU	
Dropout (p = 0.2)	

Retinal fundus images were preprocessed according to the procedure presented in^61^ to correct the different lighting and contrast conditions, thus allowing the dataset to be more uniform. Images were then normalized and standardized based on ImageNet values to further improve uniformity. Data was artificially augmented by proceeding to random horizontal and vertical flips, random rotation (0° to 360°). Data augmentation was limited to those transformations as shearing and affine transformations had a negative impact on training. For learning, the CLSA dataset was divided into a training set (70%), a validation set to access model performance during the training phase (15%) and a testing set used to independently evaluate the final model (15%). All deep learning related work was performed in python using PyTorch 1.8.0 and is available at https://github.com/cacoool/CLSA-Retina.

### Evaluating the algorithm

To assess model performance for continuous variables (age, blood pressure, cognitive functions and BMI), the mean absolute error (MAE) and the coefficient of determination (R^2^) were used. The MAE is simply defined as the difference between the expected and observed value. R^2^, to not be confounded with r^2^ which is the squared Pearson’s correlation coefficient, provides an indication of goodness of fit. According to Di Bucchianico^62^ and Barrett^63^, R^2^ represents the proportion of variance that can be explained by the independent variables in the model. It provides an indication of goodness of fit and therefore a measure of how well unseen samples are likely to be predicted by the model, through the proportion of explained variance. Best possible score is 1.0 and it can be negative (because the model can be arbitrarily worse). A constant model that always predicts the expected value of y, disregarding the input features, would get a R^2^ score of 0.0. Thus, a positive value [0, 1] of R^2^ indicates that the model is explaining a certain amount of variance based on input features and a negative value ]− ∞, 0[ indicates that the model is arbitrarily worse than simply predicting the mean value (horizontal line). For binary classification (gender and APOE4 status), area under curve (AUC) was used.

### Statistical analysis

To evaluate 95% confidence intervals (CIs) on predicted values, a non-parametric bootstrap method was used. 95% CIs on metrics (MAE, ROC AUC and R^2^) were computed with random samples (the same size as the test dataset) obtained from the test dataset. Then, a distribution of the performance metrics was obtained out of 2000 iterations to compute reported 95% CIs values.

## Supplementary Information

Supplementary Information.

## Data Availability

Data are available from the Canadian Longitudinal Study on Aging (http://www.clsa-elcv.ca) for researchers who meet the criteria for access to de-identified CLSA data.

## Code availability

Models and code that support the findings of this study are available at https://github.com/cacoool/CLSA-Retina.
