# Supplementary material for: Assessment of the predictive potential of cognitive scores from retinal images and retinal fundus metadata via deep learning using the CLSA database
Source: Sci Rep. 2022 Apr 6;12:5767. doi: 10.1038/s41598-022-09719-3 (PMC8986784; doi:10.1038/s41598-022-09719-3)
Supplement: Supplementary file 1 — Supplementary Information. [file 41598_2022_9719_MOESM1_ESM.docx]

**Assessment of the predictive potential of cognitive scores from retinal images and retinal fundus metadata via deep learning using CLSA database**

**Supplementary Information**

**Denis Corbin**^1,*^**, Frédéric Lesage**^1,2^

^1^Laboratoire d’Imagerie optique et moléculaire, Polytechnique Montréal, 2500 Chemin de Polytechnique Montréal, Montréal (Québec), Canada, H3T 1J4

^2^Institut de Cardiologie de Montréal, 5000 Rue Bélanger, Montréal (Québec), Canada, H1T 1C8

*denis.corbin@hotmail.com


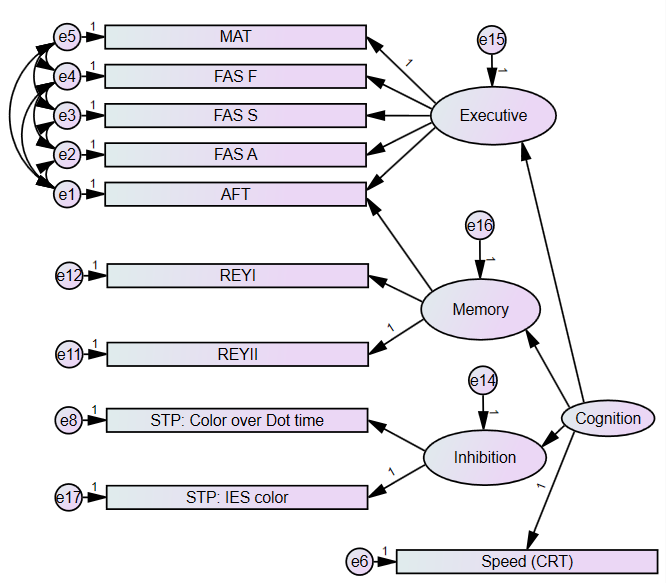


Figure 1: Accepted CFA model based on EFA and theory. No variable had a correlation factor above >0.8 in any scale, therefore linear independence criterion within a scale was respected. Executive and memory scale are based of the variables highlighted by the EFA as they agreed with theory. Inhibition scale is represented by ratios (IES [1] and color over dot time) calculated from the Stroop Test. Speed scale is directly represented by CRT test scores as it was the only test that independently measured this aspect.

|  |  | **EFA Components** | | | |
| --- | --- | --- | --- | --- | --- |
|  | **Cognitive tests** | **1** | **2** | **3** | **4** |
| Executive function | **FAS S** | 0.850 |  |  |  |
|  | **FAS A** | 0.848 |  |  |  |
|  | **FAS F** | 0.843 |  |  |  |
|  | **MAT** | 0.504 |  |  |  |
|  | **AFT** | 0.492 | 0.392 |  |  |
| Memory | **REYI** |  | 0.889 |  |  |
|  | **REYII** |  | 0.877 |  |  |
| Speed and inhibition | **Stroop ratio (Word/dot)** |  |  | 0.736 |  |
|  | **Stroop ratio (Color/dot)** |  |  | 0.678 |  |
|  | **CRT** |  |  | 0.321 |  |
| Prospective memory | **PMT** |  |  |  | -0.733 |
|  | **TMT** |  |  |  | 0.673 |
|  | **Cronbach’s alpha** | 0.799 | 0.710 | 0.237 | -0,025 |

Table 1. Grouping of cognitive constructs based on EFA. The four main principal components reported explained 60,15% of dataset’s variance. Components were extracted using principal component analysis (PCA) with rotation based on varimax with Kaiser normalization. According to computed Cronbach’s alphas, scales for components 3 and 4 are incoherent (<0.7) and will therefore be modified in CFA.

| **Criterions** | **Threshold** | **Reported value** |
| --- | --- | --- |
| (A)GFI | GFI ≥ 0.95  AGFI ≥0.90 | GFI =0.997  AGFI = 0.992 |
| RMSEA | RMSEA<0.06 | 0.026 |
| S(RMR) | S(RMR) < 0.08 | 0.000 |
| NFI | NFI > 0.95 | 0.995 |
| CFI | CFI > 0.95 | 0.995 |

Table 2: CFA models and criterions validity. Thresholds of criterions are based on [2-4]. Chi squared is not reported as it is an unreliable rejection/acceptance criterion for models representing large sample sizes [2].

|  | **Executive function** | **Memory** | **Speed** | **Inhibition** |
| --- | --- | --- | --- | --- |
| **Cronbach’s alpha** | 0.799 | 0.710 | N.A. | 0.699 |

Table 3: Internal coherence of cognitive constructs based on the accepted CFA model presented in Figure 1. The proposed CFA model shows internal coherence with Cronbach’s alphas ≥ 0.7 where applicable (more than one test for a construct).

|  | | EfficientNet  Fundus only | | | EfficientNet  Fundus and Metadata | | |
| --- | --- | --- | --- | --- | --- | --- | --- |
| (n=) | **Error**  **margin** | **Model accuracy (%)** | **Baseline accuracy (%)** | **P value** | **Model accuracy (%)** | **Baseline accuracy (%)** | **P value** |
| Age  (years) | ±1 | 19 | 9 | <0.0001 |  |  |  |
|  | ±3 | 53 | 26 | <0.0001 |  |  |  |
|  | ±5 | 77 | 42 | <0.0001 |  |  |  |
| SBP  (mmHG) | ±5 | 29 | 26 | <0.0001 |  |  |  |
|  | ±10 | 54 | 50 | <0.0001 |  |  |  |
|  | ±15 | 74 | 70 | <0.0001 |  |  |  |
| DBP  (mmHG) | ±3 | 28 | 23 | <0.0001 |  |  |  |
|  | ±5 | 45 | 38 | <0.0001 |  |  |  |
|  | ±10 | 76 | 70 | <0.0001 |  |  |  |
| BMI  (A.U.) | ±1 | 18 | 16 | 0.03 |  |  |  |
|  | ±3 | 50 | 46 | <0.0001 |  |  |  |
|  | ±5 | 73 | 70 | <0.0001 |  |  |  |
| Executive function  (A.U.) | ±3 | 29 | 27 | 0.02 | 30 | 27 | <0.01 |
|  | ±5 | 47 | 44 | <0.001 | 48 | 44 | <0.0001 |
|  | ±10 | 79 | 77 | <0.0001 | 81 | 77 | <0.0001 |
| Speed  (A.U.) | ±3 | 26 | 25 | 0.09 | 31 | 25 | <0.0001 |
|  | ±5 | 41 | 40 | 0.05 | 45 | 40 | <0.0001 |
|  | ±10 | 73 | 69 | <0.0001 | 74 | 69 | <0.0001 |
| Memory  (A.U.) | ±3 | 21 | 25 | p>0.1 | 26 | 25 | 0.06 |
|  | ±5 | 30 | 36 | p>0.1 | 37 | 36 | 0.01 |
|  | ±10 | 62 | 70 | p>0.1 | 72 | 70 | <0.01 |
| Inhibition  (A.U.) | ±3 | 29 | 27 | 0.02 | 30 | 27 | <0.01 |
|  | ±5 | 44 | 42 | <0.001 | 47 | 42 | <0.0001 |
|  | ±10 | 76 | 74 | <0.0001 | 80 | 74 | <0.0001 |
| Global cognition (A.U.) | ±3 | 26 | 25 | 0.07 | 30 | 25 | <0.0001 |
|  | ±5 | 42 | 39 | <0.001 | 46 | 39 | <0.0001 |
|  | ±10 | 72 | 70 | <0.0001 | 76 | 70 | <0.0001 |

Table 4. Accuracy on predicting variables within a fixed range. Baseline accuracy was generated by sliding a window with a size equal to the error bounds (for example, size 10 for ± 5) across the population histogram and then taking the maximum of the summed histogram counts. This provides the maximum possible ‘random’ accuracy (by guessing the centre of the sliding window corresponding to the maximum). P-values were obtained using a one-tailed binomial test (n = number of patients,) with the baseline accuracy as the chance probability of a correct prediction.

**References**

[1] J. T. Townsend and F. G. Ashby, "Methods of modeling capacity in simple processing systems," in *Cognitive theory*: Psychology Press, 2014, pp. 211-252.

[2] H. Gatignon, "Confirmatory Factor Analysis," in *Statistical Analysis of Management Data*. New York, NY: Springer New York, 2010, pp. 59-122.

[3] L. t. Hu and P. M. Bentler, "Cutoff criteria for fit indexes in covariance structure analysis: Conventional criteria versus new alternatives," *Structural Equation Modeling: A Multidisciplinary Journal,* vol. 6, no. 1, pp. 1-55, 1999/01/01 1999, doi: 10.1080/10705519909540118.

[4] T. A. Brown, *Confirmatory factor analysis for applied research*. Guilford publications, 2015.
